# Nuclear Localization of Apolipoprotein E4: A New Trick for an Old Protein

**DOI:** 10.23937/2378-3001/1410067

**Published:** 2017-07-31

**Authors:** Troy T Rohn, Zachary D Moore

**Affiliations:** Department of Biological Sciences, Boise State University, USA

**Keywords:** Apolipoprotein E4 (ApoE4), Proteolysis, Alzheimer’s disease, Nucleus, Transcription, Dementia, Receptor-mediated endocytosis, LDL receptor-related protein

## Abstract

One of the most important genetic risk factors for late-onset Alzheimer’s Disease (AD) is harboring the *ApoE4* allele. Much is known regarding the functions of the ApoE4 protein including cholesterol transport in the CNS and a critical role in clearing beta-amyloid deposits in the AD brain. However, recent studies demonstrating the nuclear localization suggest a novel function beyond the classical known actions of ApoE4. The purpose of the current review is to examine how this secreted protein traffics to the nucleus and to discuss possible outcomes of nuclear localization in the CNS. It is suggested that proteolytic fragmentation of ApoE4 is a key step leading to nuclear localization and the outcome of this event is to initiate transcription of various genes involved in inflammation and cell death. Therefore, the nuclear localization and induction of gene expression may provide a link between harboring the *ApoE4* allele and enhanced dementia risk observed in AD.

## Apolipoprotein E Structure and Function in the CNS

Human ApoE is polymorphic with three major isoforms, ApoE2, ApoE3, and ApoE4, which differ by single amino acid substitutions involving cysteine-arginine replacements at positions 112 and 158 [1]. ApoE is synthesized as a precursor protein consisting of 317 amino acids containing an 18 amino acid leader sequence. Cleavage of this linker sequence gives the mature form of ApoE consisting of 299 amino acids and a molecular weight of 34 kDa [2]. Structurally, ApoE consists of two major domains that are N-terminal (20 kDa) and C-terminal (10 kDa), separated by a short hinge region [3]. The hinge region of ApoE contains multiple protease sensitive sites and evidence now suggests that ApoE4 is much more susceptible to proteolysis than E3 or E2 and this in fact may link the enhanced risk of harboring *ApoE4* to dementia observed in AD (see [4] for recent review).

In the CNS, ApoE is synthesized and secreted by astrocytes [5] and, to a lesser extent, by microglia [6], oligodendrocytes [7], and neurons following injury [8]. Functionally, ApoE is a major cholesterol carrier that supports lipid transport and injury repair in the brain. In this manner, ApoE delivers cholesterol to neurons via several different ApoE receptors that make up collectively, the Low-Density Lipoprotein Receptor (LDLR) family [9,10]. It has been proposed that because ApoE is the major cholesterol transporter in the brain (see below) and therefore is essential for synaptogenesis in neurons, then ApoE-isoform-dependent differences in these processes may negatively impact synaptic plasticity or recovery of neurons from neurodegeneration as might occur in AD [11,12].

## *ApoE4* as a Risk Factor for Alzheimer’s Disease

Human ApoE is polymorphic with three major isoforms, ApoE2, ApoE3, and ApoE4, which differ by single amino acid substitutions involving cysteine-arginine replacements at positions 112 and 158 [1]. The frequency of the three polymorphic alleles is 8.4% for *E2*, 77.9% for *E3* and 13.7% for *E4* [13]. Harboring the *ApoE3* allele is believed to neither increase nor decrease one’s risk of AD, while having the *E2* form may decrease one’s risk. In contrast, inheritance of one copy of the *ApoE4* allele increases disease risk fourfold, while two copies raises the risk tenfold [14]. It is noteworthy that 65–80% of all AD patients have at least one *ApoE4* allele [13,15]. How the ApoE4 protein enhances dementia risk has been the subject of intense study and the prevailing view is ApoE4 is less effective in promoting the clearance of beta-amyloid deposits. Thus, both pathological and neuroimaging studies have indicated that beta-amyloid deposition occurs to a greater extent and earlier in the disease process in *ApoE4* carriers [16–18].

The exact mechanism by which ApoE enhances clearance of beta-amyloid plaque loads is unclear [19–21]. It has been proposed that ApoE can bind to beta-amyloid and undergo endocytosis via the astrocyte low-density Lipoprotein Receptor-Related Protein (LRP) [22,23]. A recent study has demonstrated a differential regulation of beta-amyloid clearance by isoforms of ApoE, with ApoE3 being more efficient in this process than ApoE4 [24]. Therefore, harboring the *ApoE4* allele may lead to a decreased ability to clear beta-amyloid deposits and accelerate the disease process in AD. An alternative hypothesis is based on a greater propensity of ApoE4 to undergo proteolysis and potential loss of function as compared to ApoE3 or E2 [4]. In this regard, ApoE4 is more susceptible to proteolysis compared to ApoE3 and E2 [25] and ApoE4 fragments (14–20 kDa) have been identified in the AD brain [26]. Further support of this hypothesis came from Bien-Ly, et al. who crossed an AD mouse model with mice that express C-terminal-truncated ApoE4 and showed these mice had a lower affinity for beta-amyloid and a reduced ability to clear beta- amyloid [27].

## Nuclear Localization of ApoE4

**Figure 1:**
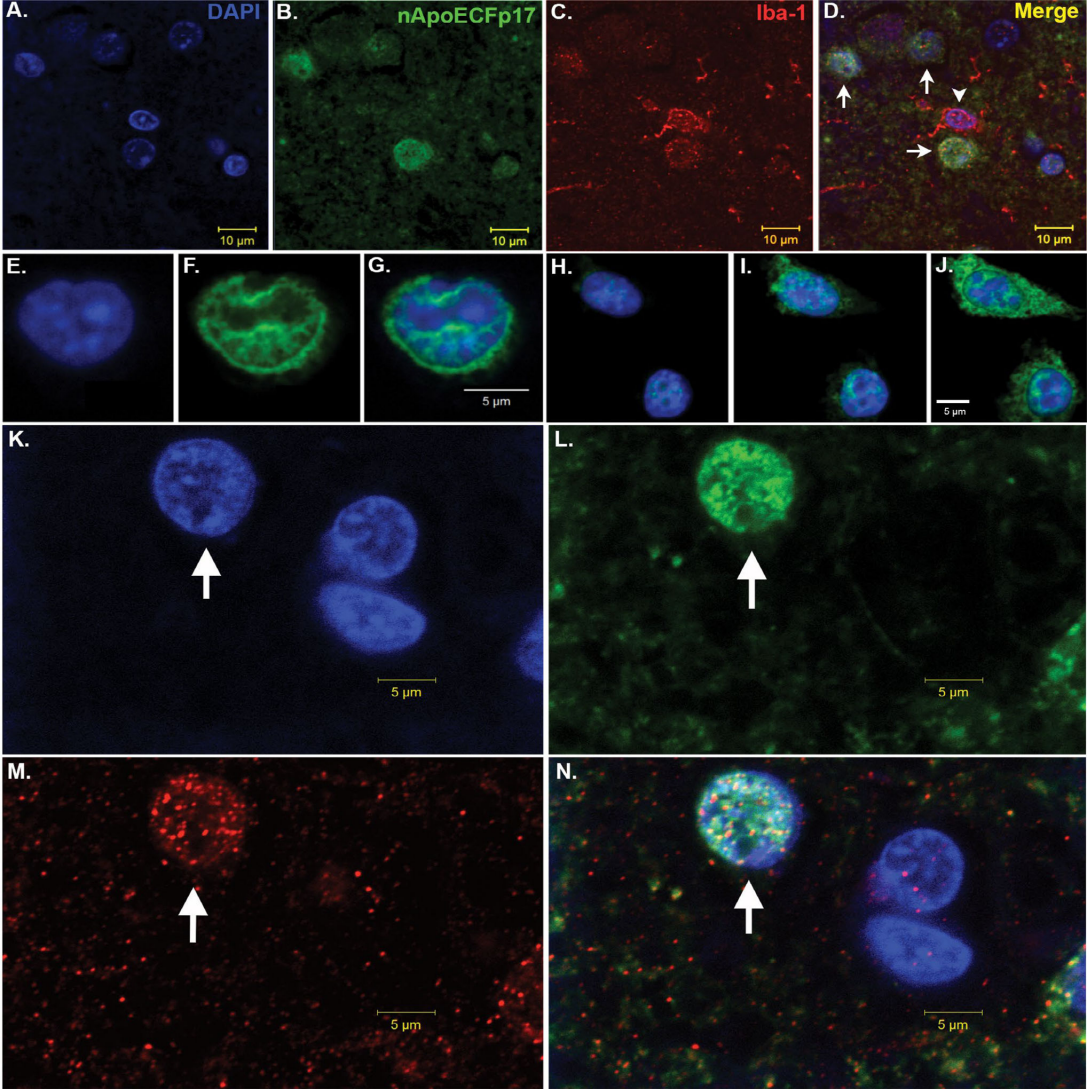
Nuclear localization of an amino-terminal fragment of ApoE within microglia of the AD brain (A–D) Representative images from confocal immunofluorescence in AD utilizing the nuclear stain, DAPI, (blue, A), nApoECFp17, an antibody that specifically detects a 17 kDa amino-terminal fragment of ApoE4 (green, B), the microglial specific marker, Iba1 (red, C), with the overlap image shown in (D). Note the colocalization of the two markers with DAPI in Panel D (arrows), however there was one Iba1-labeled microglia that did not label with the nApoECFp17 antibody (arrowhead); (E–J) Two representative sets of images showing nuclear localization of in the microglial cell line, BV2, following exogenous treatment with the ApoE4_1–151_ fragment. BV2 microglial cells were placed on glass chamber slides in normal growth media and treated for 24 hours with the His-tagged ApoE4_1–151_ fragment (50 µg/ml). Following treatment, cells were fixed and immunocytochemistry was carried out using an anti-His rabbit secondary antibody. Double-label immunofluorescence confocal z-stacks were acquired with an anti-His antibody to detect ApoE4_1–151_ (green, Panels F and I) together with DAPI (blue, E and H). The merged images indicated the strong nuclear and cytoplasmic presence of the amino-terminal fragment following extracellular incubation of BV2 cells (Panels G and J); (K–N) Representative images from confocal immunofluorescence in the human AD brain utilizing the nuclear stain, DAPI, (K), nApoECFp17 (L), the microglial specific marker, Iba1 (M), with the overlap image shown in (N). Note the strong nuclear localization of the nApoECFp17 antibody (arrow) within labeled microglia.

**Figure 2:**
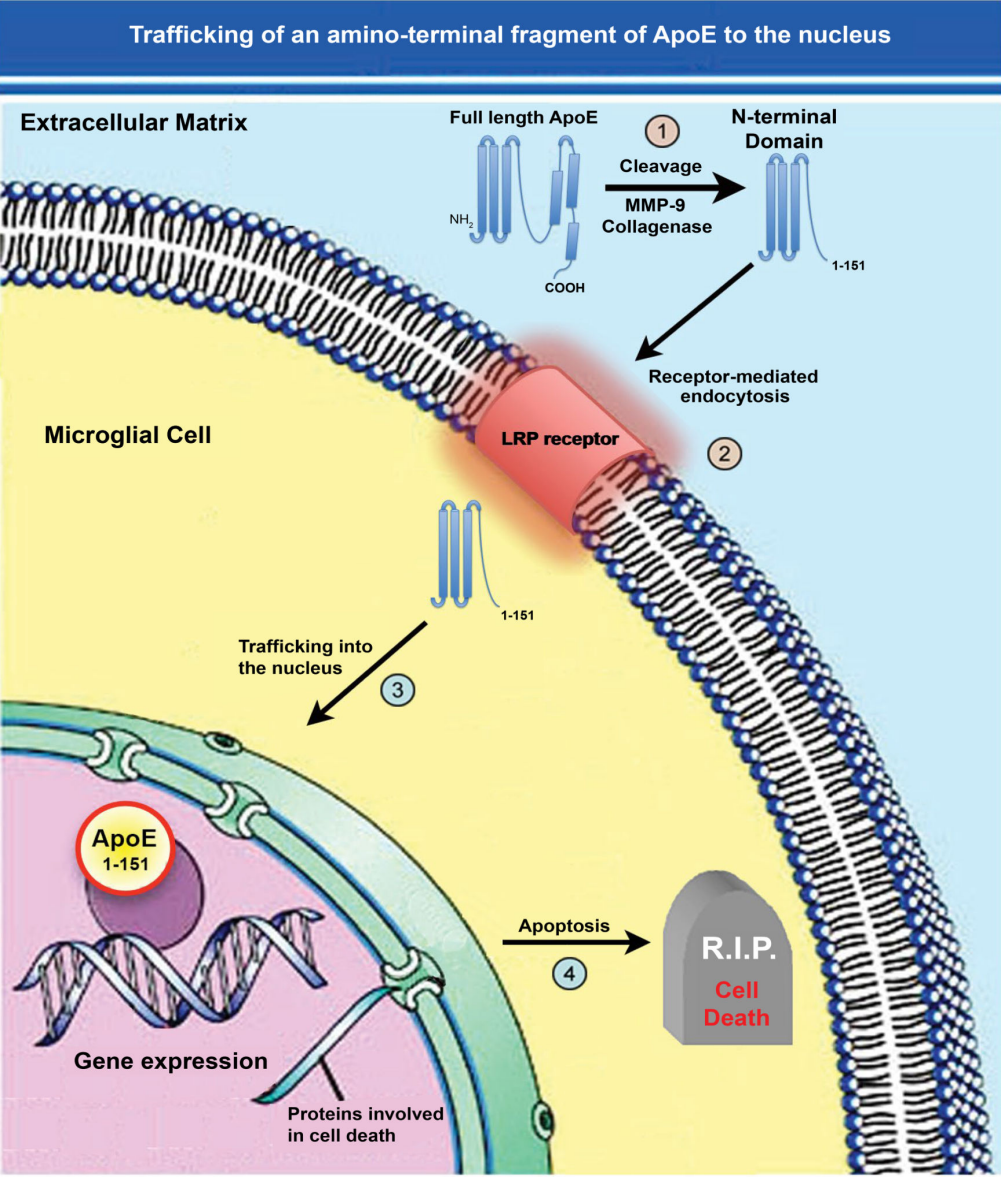
Trafficking of an amino-terminal fragment of ApoE4 to the nucleus (1) ApoE4 may promote the pathogenesis underlying AD following cleavage in the extracellular compartment by collagenase or MMP-9; (2) The generation of this fragment by then lead to specific uptake into glial cells such as microglia, through the LRP receptor via Receptor-Mediated Endocytosis (RME); (3) This fragment then traffics to the nucleus and due to its’ small size, may passively diffuse through nuclear pore complexes unassisted; (4) Once inside the nucleus, this amino-terminal fragment of ApoE4 may act as a transcription factor leading to the expression of genes that promote cell death or enhance inflammation. In addition to this scheme involving an amino-terminal fragment of ApoE4 generated extracellularly, a similar pathway may be involved for full-length ApoE4 cleaved by unknown proteases within glial cells that normally express ApoE. Cellular entry through RME would lead to residence of ApoE fragments within endosomes and eventually lysosomes (not shown). How ApoE could escape degradation once in lysosomes is currently not known.

Our lab was interested in determining whether proteolysis of ApoE4 was an important event occurring in AD and to address this, we synthesized a site-directed cleavage antibody that specifically recognized an amino-terminal fragment of 17 kDa (p17) following cleavage after D151 of the mature, full-length form of ApoE4 [28]. This antibody, which we termed the amino-terminal ApoE4 cleavage fragment antibody (nApoECFp17 antibody) was highly specific for this fragment following cleavage of full-length ApoE4 with collagenase or MMP-9 and showed no immunoreactivity to the full-length, 34 kDa form of the protein [28]. *In situ*, we demonstrated widespread labeling of this antibody in the AD brain and most surprising, strong localization within microglia (Figure 1). We further showed that a recombinant produced fragment of ApoE4_1–151_ was taken up by the microglia cell line, BV2, following extracellular treatment and trafficked to the nucleus causing significant toxicity. Interesting, an identical ApoE3_1–151_ fragment was used as a comparison and although this fragment was taken up into the cytoplasm, no nuclear localization was evident and furthermore, there was no evidence for cytotoxicity [28]. The only difference between these two fragments was the presence of a cysteine residue at position 112 for the ApoE3_1–151_ fragment in lieu of an arginine in ApoE4_1–151_. This suggested that nuclear localization of ApoE4_1–151_ and eventual cell death could be linked events. Taken together, our results shed light on a potential novel pathway for ApoE4 whereby localization within the nucleus may lead to gene expression of cell death pathways. Figure 2 summarizes a putative pathway of nuclear localization of an amino-terminal fragment of ApoE4 leading to gene expression and cell death.

## Structural Differences in ApoE4 Versus ApoE3 Amino-Terminal Fragments

**Figure 3:**
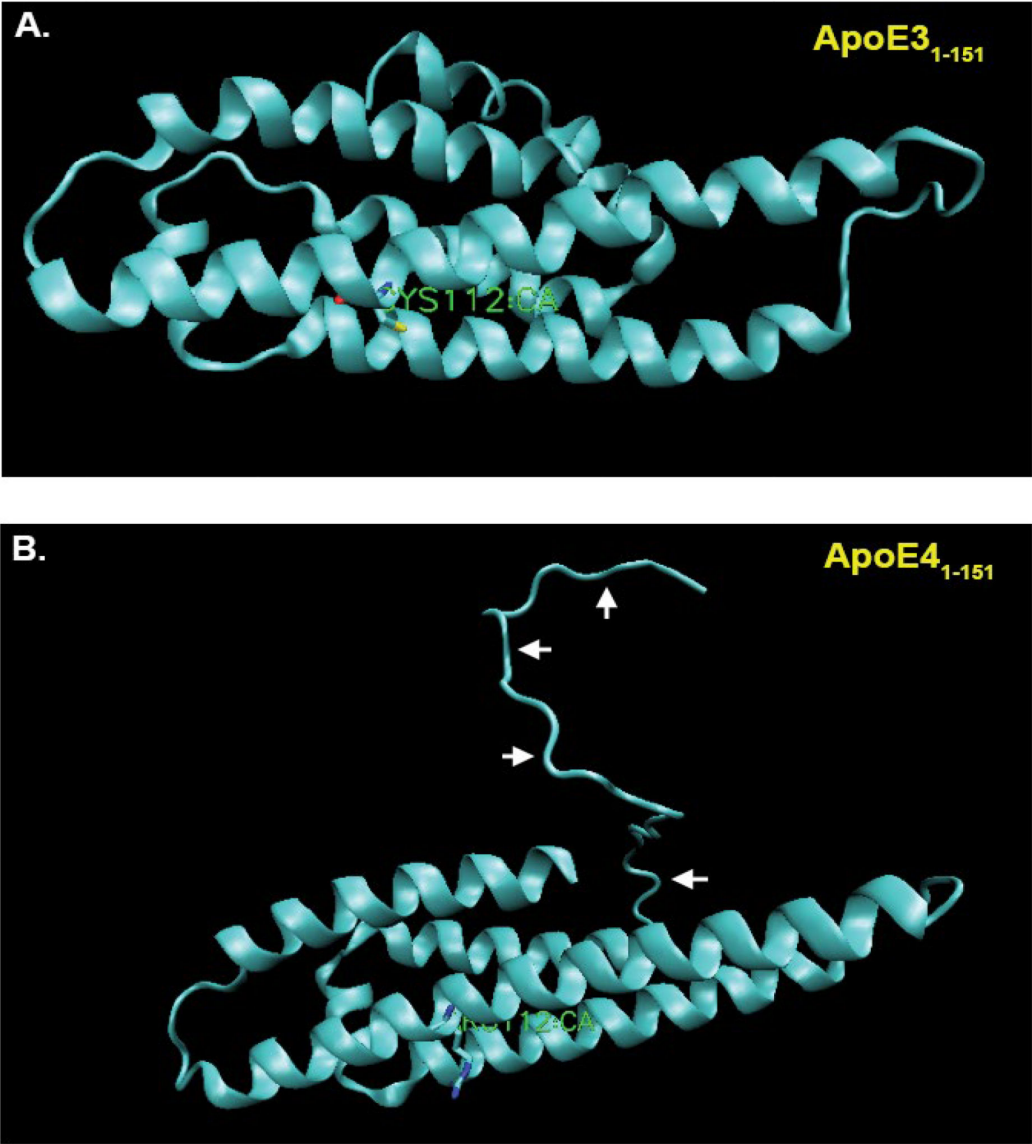
Predictive structural modeling of amino-terminal fragments of ApoE3 and E4 (A) VMD representation of the MPI-predicted structure of the aminApoE3_1–151_ with listed cysteine 112 residue shown in green. Note alpha-helical secondary structural predicted by this amino acid sequence; (B) VMD representation of the MPI-predicted structure of the aminApoE4_1–151_ with listed arginine 112 residue shown in green. In this case, note the “hook” region of the amino-terminus that extends outward from the amino-terminal fragment (arrows).

Our results supported the nuclear localization and cytotoxicity of ApoE4_1–151_ as compared to ApoE3_1–151_, even though these fragments only differ by a single amino acid. What could account for such a difference? Given that the substitution responsible is only one residue, there may be structural or functional variation between the two amino-terminal fragments with consequential downstream effects. Current predictive modeling techniques can provide great insight into the inherent properties of proteins, and the potential resulting interactions. One such program, the MPI Bioinformatics Toolkit, begins with sequence alignment drawing data from two global protein databases that include RCSB PDB (Research Collaboratory for Structural Bioinformatics Protein Data Bank) and SCOPe (Structural Classification of Proteins-extended) to then assign interspecies proteome homology to an input amino acid sequence. The three-dimensional modeling it generates is based on protein structures that have been determined through X-ray crystallography, NMR spectroscopy, and cryo-electron microscopy [29]. With this data, an algorithm cross-references the protein database information to yield a file containing the predicted tertiary structure of the protein that may be put into any visualization software that recognizes .pdb files. For the images generated in Figure 3, Visual Molecular Dynamics (VMD) was used to visualize the structures [30]. In the case of the two sequences of the 151 amino acid N-terminal fragments of ApoE3 and ApoE4 entered in to the MPI toolkit, 100% matches to homologous proteins contained within the databanks were found. The proteins bearing PDB identification numbers 1NFN and 1GS9 were used as templates for ApoE3 and ApoE4, respectively, to generate models (Verderame, J.R., Kantardjieff, K., Segelke, B., Weisgraber, K., Rupp, B., 2003; Apolipoprotein E4, 22K domain. PDBID: 1GS9. DOI: 10.2210/pdb1gs9/pdb. Rupp, B., Parkin, S., 1997; Apolipoprotein E3 (*ApoE3*). PDBID: 1NFN. DOI: 10.2210/pdb1nfn/pdb). At first glance the secondary structure of either of ApoE3_1–151_ and ApoE4_1–151_ amino-terminal fragments appears similar to full-length ApoE that is both fragment and full-length forms adopt predominantly alpha-helical secondary structure. However, examining the tertiary differences between ApoE3_1–151_ and ApoE4_1–151_ amino-terminal fragments appears to reveal subtle differences: Although the ApoE3_1–151_ and ApoE4_1–151_ amino-terminal fragments differ by only a C112R substitution, the tertiary structure of the E4 fragment of the protein appears to have taken on an altered tertiary structure based on modeling predictions (Figure 3). As seen in Figure 3B, the first 24 residues originating from the N-terminal of ApoE4_1–151_ do not take on any secondary structure, instead producing a potential “hook” region that extends outward from the fragment. In contrast, in the ApoE3_1–151_ fragment, this region adopts a predictive alpha-helical structural that remains folded (Figure 3A). It is possible that this “hook” domain of ApoE4_1–151_ is the difference that allows for nuclear localization and subsequent toxicity. Although speculative at this point, these potential structural differences could provide impetus for future studies.

## Other Studies Supporting Nuclear Localization of ApoE

In addition to our findings several previous studies have also documented the nuclear localization of the ApoE protein. For example, recently, Theendakara, et al. demonstrated a role for ApoE as a DNA binding protein and transcriptional regulator of multiple genes [31]. Using a combination of neural cell lines, skin fibroblasts from AD patients, and ApoE targeted replacement mouse brains, the authors eloquently showed nuclear localization of ApoE4 and binding to double-stranded DNA with high affinity that was in the range of known transcription factors (~10 nM) [31]. A key finding in this study was evidence demonstrating direct binding of ApoE4 and E3 to the SirT1 promoter region [31]. Moreover, the authors were able to identify 76 genes activated by ApoE4 and included among them genes involved in programmed cell death as well as three genes linked to inflammation [31]. Gene expression of proteins involved in programmed cell death would support our findings showing significant cytotoxicity following nuclear localization of ApoE4_1–151_ in BV2 cells [28]. In terms of the transcriptional role of ApoE4 fragments versus full-length ApoE4, it is unknown whether different genes will be regulated. However, it is important to note that in the study by Theendakara, et al., the authors expressed full-length ApoE4 in cells, but did not actually determine whether the transcriptional regulation in their cell model was occurring by full-length ApoE4 or ApoE4 that may have been proteolyses in the cytoplasm by an unknown protease. Further studies will be necessary to see if there are different patterns of gene expression elicited between full-length and fragmented ApoE4.

Levros, et al. demonstrated the binding and repressive activities of ApoE4 and E3 on the human ApoD promoter in hepatic and glioblastoma cell lines. In addition, over expression of ApoE3 and E4 isoforms but not ApoE2 significantly inhibited the ApoD promoter activity in U87 cells suggesting that ApoE can regulate ApoD gene expression [32]. Taken together, these studies support the hypothesis that ApoE can possibly function as a transcription factor, highlighting a novel role for this protein.

Other studies have also supported the nuclear localization of ApoE including Kim, et al. who used Green Fluorescent (GFP) fusion proteins to demonstrated that all three ApoE isoforms could be detected in the nucleus of CHO cells [33]. Interesting, in this study the level of ApoE-GFP in the nucleus increased following serum starvation, a cellular stress signal [33]. In a parallel study, Quinn, et al. demonstrated nuclear pools of ApoE in human fibroblasts under conditions of serum-starvation-induced growth arrest [34]. The first documented nuclear localization of ApoE came from Panin, et al. who showed immunoreactivity in various rat tissue cells [35]. The authors concluded that ApoE could be involved in the regulation of transcriptional activity of chromatin.

## Mechanism of Nuclear Trafficking of ApoE

A corollary to the nuclear localization of ApoE is how it traffics to the nucleus. Our previous results uncovered a potentially novel mechanism by which ApoE4 is cleaved by MMP-9, and the subsequent amino-terminal fragment is taken up by cells and traffics to the nucleus [28]. Examination of the sequence of the immunogenic peptide of ApoE4 used to synthesize the nApoECF antibody indicates the epitope is near position D151 of the full-length ApoE protein. Cleavage by MMP-9 after this aspartic residue would generate an approximate 17 kDa amino-terminal fragment. Interesting, the ApoE protein has several domains including the C-terminal domain that is known to bind to cholesterol and beta-amyloid, an amino-terminal domain that primarily includes the LDL receptor-binding region (residues 134–150), and a hinge region connecting the two domains that contains a number of putative proteolytic cleavage sites [36,37]. Coincidently, the cleavage site recognized by the nApoECF antibody coincides directly with the low-density Lipoprotein Receptor Related Protein (LRP)-binding region. Therefore, we hypothesize that cleavage of full-length ApoE4 at position 151 generates an amino-terminal fragment that may interact with high affinity to LRP, is taken up through receptor-mediated endocytosis, traffics to the nucleus, and thereby alters gene expression. We hypothesize that it is the LRP receptor expressed on the cell surface of microglia that is responsible for mediating the uptake of ApoE_1–151_ due to the fact that LRP is the major receptor for lipid-poor forms of ApoE [38]. The lipid-binding region of ApoE is located within the C-terminal domain (residues 244–272) [37], and thus ApoE_1–151_ by lacking this domain would be lipid free. In addition, the LRP/LDL receptor-binding domain of ApoE (residues 134–150) [36] corresponds exactly to the C-terminal end of ApoE_1–151_ to which our nApoECF antibody recognizes and labels nuclei in the AD brain. Therefore, we hypothesize that this fragment may bind with high affinity to LRP and is readily taken up by cells expressing this receptor (Figure 2). However, one problem with this hypothesis is the mechanism by with ApoE amino-terminal fragments can escape the endosomal pathway and degradation by specific enzymes in the lysosome is currently unclear. This question will require further investigation.

Once residing in the cytoplasm the next question is how does ApoE traffic to the nucleus? For full-length ApoE having a molecular weight of 34 kDa would suggest that perhaps it could passively diffuse through nuclear pore complexes unassisted. Previous studies have shown that proteins greater than 50 kDa require an active transport pathway largely mediated by importins [39,40]. Additionally, ApoE could be transported into the nucleus by binding to specific nuclear proteins through their weak polybasic domains that could serve as potential nuclear signaling sequences [32,33]. Again, further studies will be necessary to determine the exact mechanism of nuclear penetration.

## Concluding Remarks

Harboring the *ApoE4 a*llele represents the most significant genetic risk factor for late-onset AD. For a protein with fairly innocuous actions in terms of it’s’ ability to shuttle cholesterol around in the CNS, how inheriting this gene leads to an increase in dementia risk has been the subject of intense study. Our current understanding of how this gene translates to enhance risk appears to revolve around, at least in part, beta-amyloid clearance and enhanced proteolysis of ApoE4 leading to a loss of function. However, recent studies have shed light on a potential new mechanism by which ApoE4 may be linked to AD. Proteolysis of ApoE4, which occurs to a greater frequency than with ApoE2 or E3, produces an amino-terminal fragment that is taken up by neurons and glial cells, traffics to the nucleus, and promotes cell death by activation of gene transcription. In this manner, ApoE4 and associated fragments may be serving as transcription factors leading to the expression of genes involved in programmed cell death and inflammation. These findings suggest a novel role for ApoE4 beyond its’ classical actions related to lipid trafficking in the CNS. Future studies should help confirm this potential novel role for ApoE4 and perhaps lead to a better understanding as to why inheritance of this gene enhances AD risk.
